# Enhanced reduction of polymicrobial biofilms on the orthodontic brackets and enamel surface remineralization using zeolite-zinc oxide nanoparticles-based antimicrobial photodynamic therapy

**DOI:** 10.1186/s12866-021-02324-w

**Published:** 2021-10-07

**Authors:** Maryam Pourhajibagher, Abbas Bahador

**Affiliations:** 1 Dental Research Center, Dentistry Research Institute, Tehran University of Medical Sciences, Tehran, Iran; 2 Oral Microbiology Laboratory, Department of Microbiology, School of Medicine, Tehran University of Medical Sciences|, Tehran, Iran; 3 Fellowship in Clinical Laboratory Sciences, BioHealth Lab, Tehran, Iran

**Keywords:** Orthodontic brackets, Remineralization, Zinc oxide, Antimicrobial photodynamic therapy, Natural zeolite

## Abstract

The aim of this study was to evaluate the anti-biofilm and anti-metabolic activities of zeolite-zinc oxide nanoparticles (Zeo/ZnONPs)-based antimicrobial photodynamic therapy (aPDT) against pre-formed polymicrobial biofilms on the orthodontic brackets, as well as, assess the remineralization efficacy on polymicrobial biofilms induced enamel lesions. Following synthesis and characterization of Zeo/ZnONPs, cell cytotoxicity, hemolytic effect, and intracellular reactive oxygen species (ROS) production were determined. The anti-biofilm and anti-metabolic activities of aPDT using different concentrations of Zeo/ZnONPs were investigated. Microhardness tester and DIAGNOdent Pen were used to evaluate the changes of remineralization degree on the treated enamel slabs duration 1 and 3 months. No significant cytotoxicity and erythrocyte hemolysis were observed in treated cells with Zeo/ZnONPs. When irradiated, suggesting that the Zeo/ZnONPs were photoactivated, generating ROS and leading to reduce dose-dependently the cell viability and metabolic activity of polymicrobial biofilms. Also, the enamel surface microhardness value of exposed enamel showed a steady increase with the concentration of Zeo/ZnONPs. No statistically significant differences were shown between aPDT and sodium fluoride varnish as the control group. Overall, Zeo/ZnONPs-based aPDT with the greatest remineralization efficacy of enamel surface can be used as an anti-biofilm therapeutic method, which is involved with their potent ability to produce ROS.

## Background

Fixed orthodontic appliances may change the oral ecosystem via increasing the number of cariogenic microorganisms, accumulation of plaque, decreased plaque pH, increasing the risk of enamel demineralization, and development of gingival inflammation [1–3]. Demineralization around the orthodontic brackets and the emergence of white spot lesions (WSLs) may lead to the development of decay in patients who do not cooperate with oral hygiene, despite modern advances in the preventive methods of dental caries [4, 5].

According to the literature, *Streptococcus* spp. have the best capacity to adhere and form biofilms on the orthodontic brackets [6]. Moreover, the amount of *Lactobacillus acidophilus* as the source of acid to demineralizes the enamel increases [7]. As previously reported, treatment with orthodontic appliances enhances *Candida albicans* colonization by their ability to form biofilms [8]. Although the recent use of self-ligating brackets in orthodontics has contributed to decreasing plaque accumulation, salivary calculus accumulation over the sliding clip mechanism and into the horizontal archwire slot can disrupt the function of these brackets [9, 10].

In recent years, antimicrobial photodynamic therapy (aPDT) has been developed as an established adjuvant therapeutic approach being readily used in dentistry to improve oral health care and control the oral cavity’s microbial load [11–13]. During aPDT, a light source (such as halogen lamps, laser, or light-emitting diode) with used in dentistry to improve oral health care and control the oral cavity’s microbial load specific wavelength activates some types of drugs, called photosensitizer in the presence of oxygen, producing reactive oxygen species that induce cell death [14, 15]. As previously reported, it is far from expected that resistant bacterial strains could be expanded due to the repeated uses of aPDT due to the nonspecific nature of its underlying antimicrobial mechanism [16].

As previous studies reported zinc oxide nanoparticles (ZnONPs), belonging to the family of semiconducting metal oxide, are used as photosensitizers in aPDT [17–20]. It is reported that, by increasing the particle size of ZnO in the nanometer range, the surface energy increases, nanoparticles accumulate based on the Van der Waals forces and/or other interactions, and eventually leads to a decrease in the stability, mechanic strength, and hence of the ZnO activity and efficiency [21, 22]. To improve the biostability of ZnONPs, the attachment hosts, supports, and stabilizer materials with high surface area and pore volume are used to overcome the limitations [23, 24].

Among the various supports, zeolites (Zeos) are the porous crystalline silicates with a complex crystallographic structure, as well as, high stability and surface area. They have a highly regular and open microporous structure which formed by a three-dimensional network of [SiO_4_]^4−^ and [A1O_4_]^5−^ tetrahedral that are connected with each other. On the other hand, Zeo could have enhanced the uptake amount of some photosensitizers by target cells [25]. In this study, ZnONPs are loaded on Zeo to realize the synergistic effect, enhance their photocatalytic activity, and prevent their agglomeration. Moreover, the Zeo crystal framework is used to improve the management of ZnONPs, uniform distributions, and increase the antimicrobial activity of ZnONPs.

Also, according to the data obtained after a comprehensive literature review, no study exists to evaluate the anti-biofilm activities of Zeo/ZnONPs-based aPDT. Therefore, this ex vivo study aimed to characterize and assess the anti-biofilm growth potencies and anti-metabolic activities of natural Zeo/ZnONPs-based aPDT against preformed polymicrobial biofilms on the orthodontic brackets, as well as, evaluate the remineralization efficacy of Zeo/ZnONPs-based aPDT on polymicrobial biofilms induced enamel lesions.

## Results

### Confirmation of Zeo/ZnONPs synthesis

**Fig. 1:**
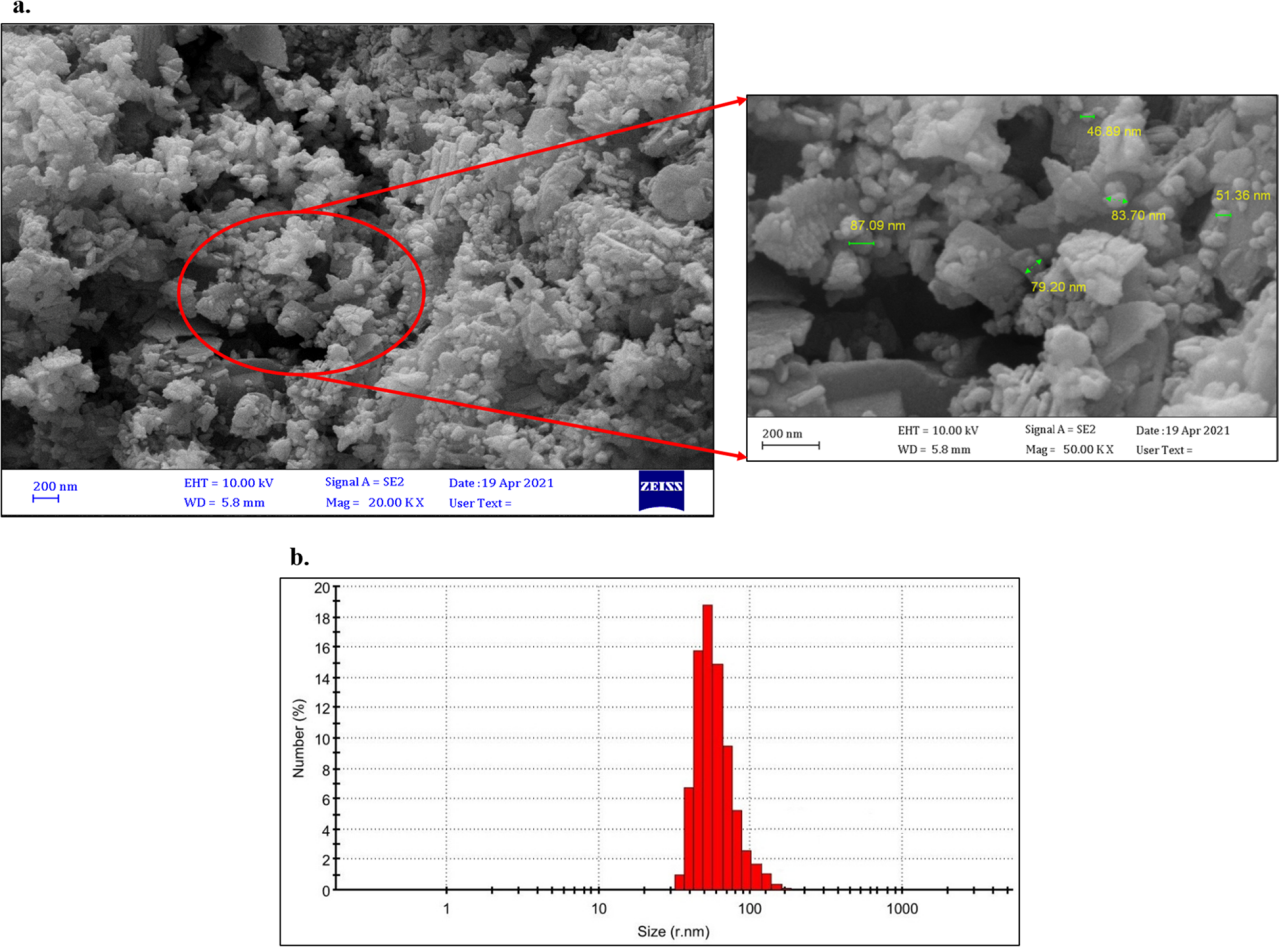
**a** FESEM image of Zeo/ZnONPs (Scale bar = 200 nm; Mag = 20.00 KX), **b** Particle size of Zeo/ZnONPs

Figures 1a demonstrate granular shapes of Zeo/ZnONPs which are confirmed by FESEM, indicating the presence of ZnONPs on the smooth surface and cubic structure of Zeo. It can be clearly seen in Fig. 1b that the particle size of Zeo/ZnONPs is in the range of 34.03–110.1 nm and an average diameter of 52.85 nm.

**Fig. 2:**
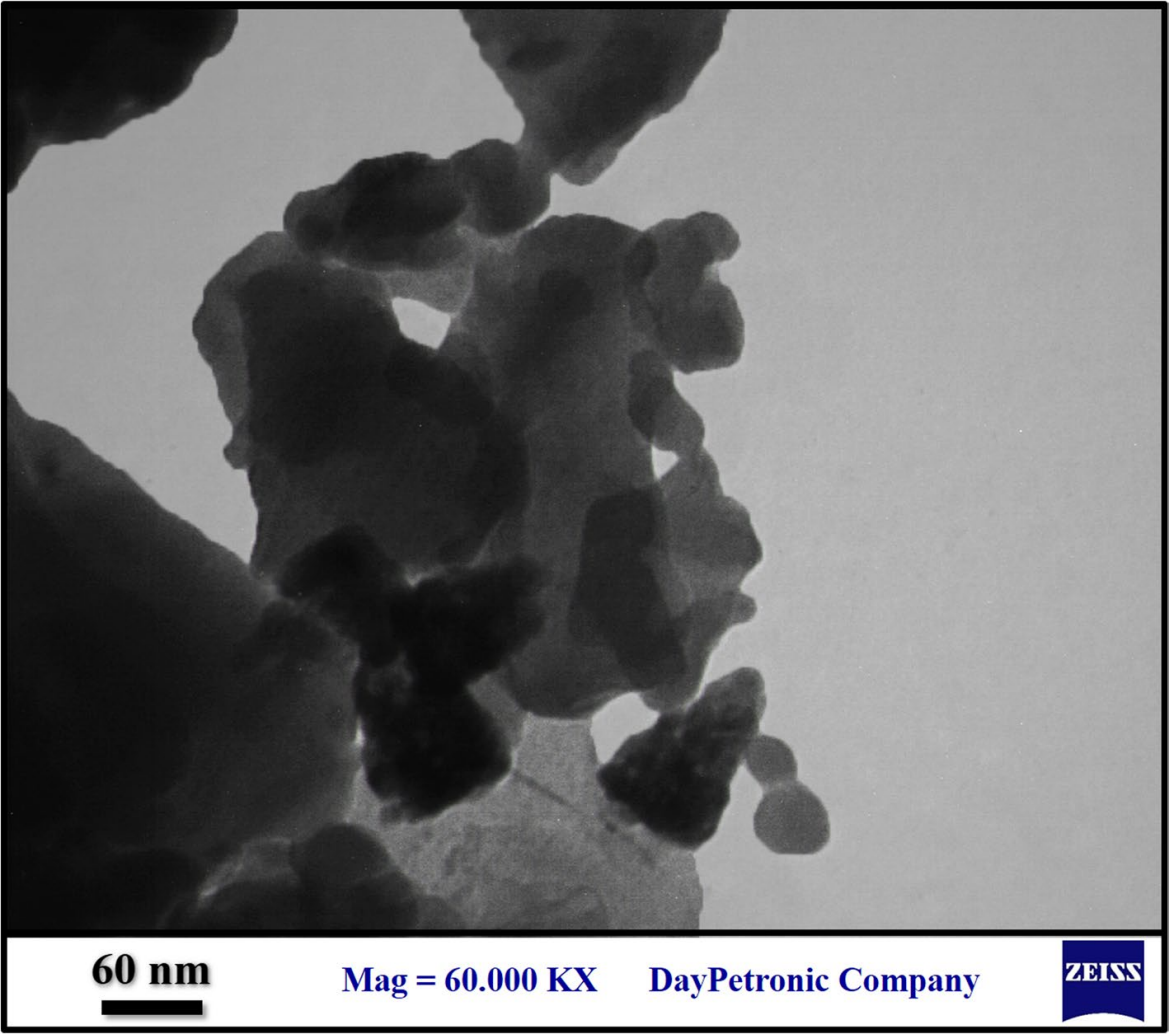
TEM image of Zeo/ZnONPs (Scale bar = 60 nm)

The presence of ZnONPs attached in Zeo is shown in the TEM image of Zeo/ZnONPs (Fig. 2).

**Fig. 3:**
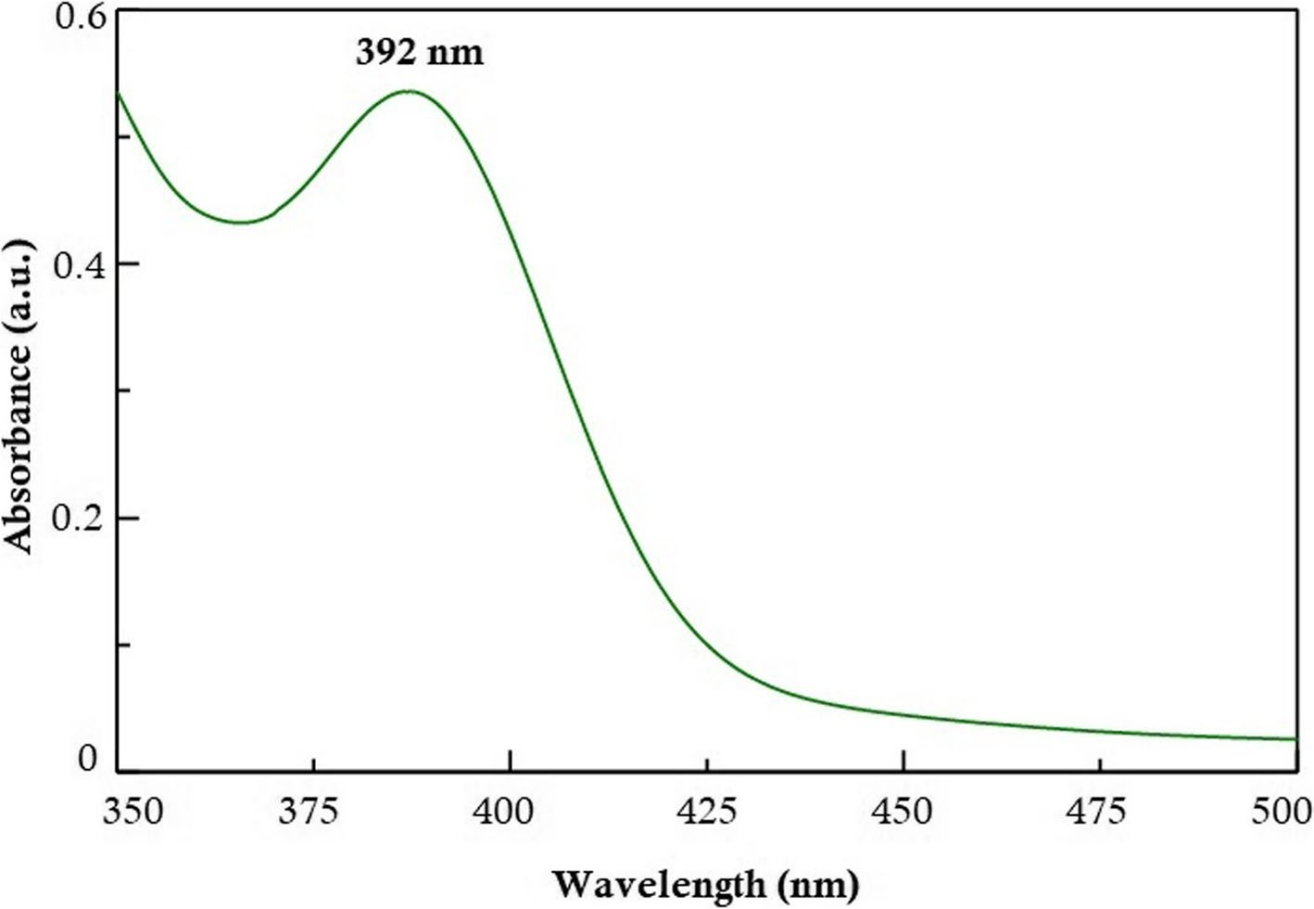
UV–visible spectrum of Zeo/ZnONPs

The UV–visible spectrum of Zeo/ZnONPs aqueous solution is presented in Fig. 3. As shown, the absorption spectrum of Zeo/ZnONPs is broadband with a maximum absorbance peak at a wavelength ~ 392 nm.

**Fig. 4:**
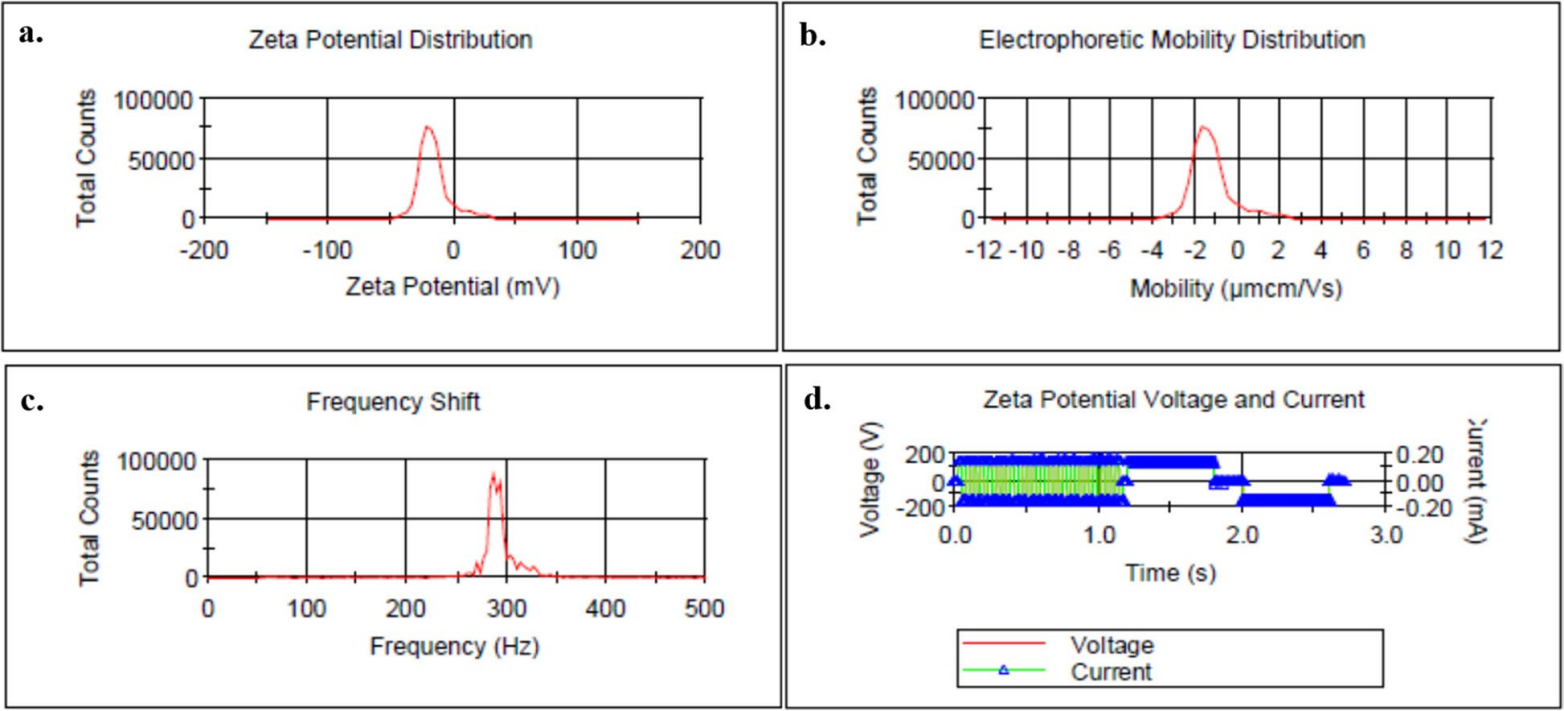
Characterization of Zeo/ZnONPs; **a** Zeta potential distribution, **b** Electrophoretic mobility distribution, **c** Frequency shift, **d** Effective voltage

According to the results in Fig. 4, the mean of zeta potential distribution of Zeo/ZnONPs was − 15.9 mV (a). The electrophoretic mobility distribution, frequency shift, and effective voltage of Zeo/ZnONPs were − 1.249 μmcm/Vs (b), 283 Hz (c), and 140 v (d), respectively.

**Fig. 5:**
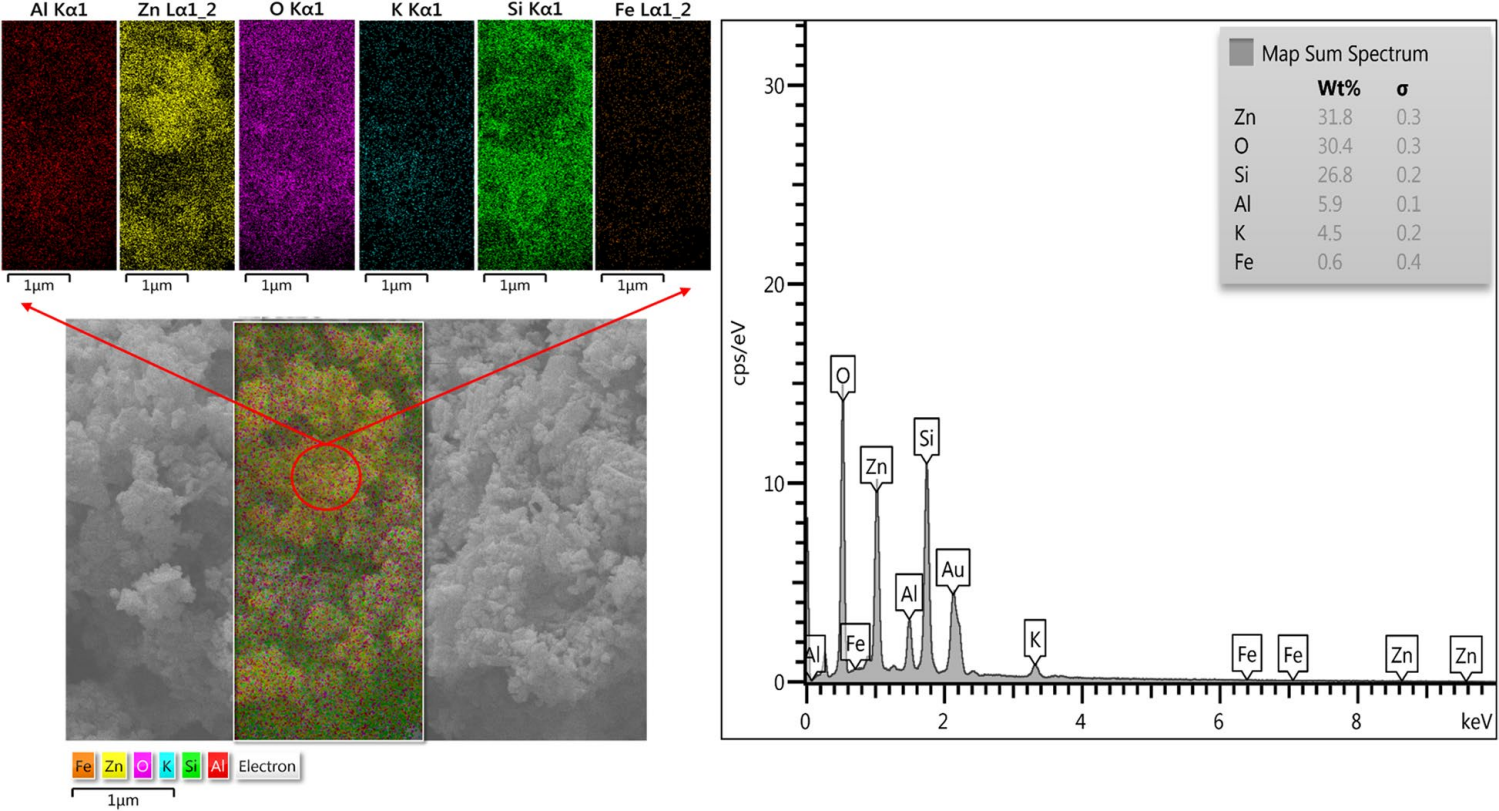
Elemental mapping of Zeo/ZnONPs

The elemental mapping of Zeo/ZnONPs observed in Fig. 5 shows a homogeneous distribution of the mentioned elements, as well as, percentage of each element over the entire nano-photosensitizer.

### Hemolysis effect of Zeo/ZnONPs

**Fig. 6:**
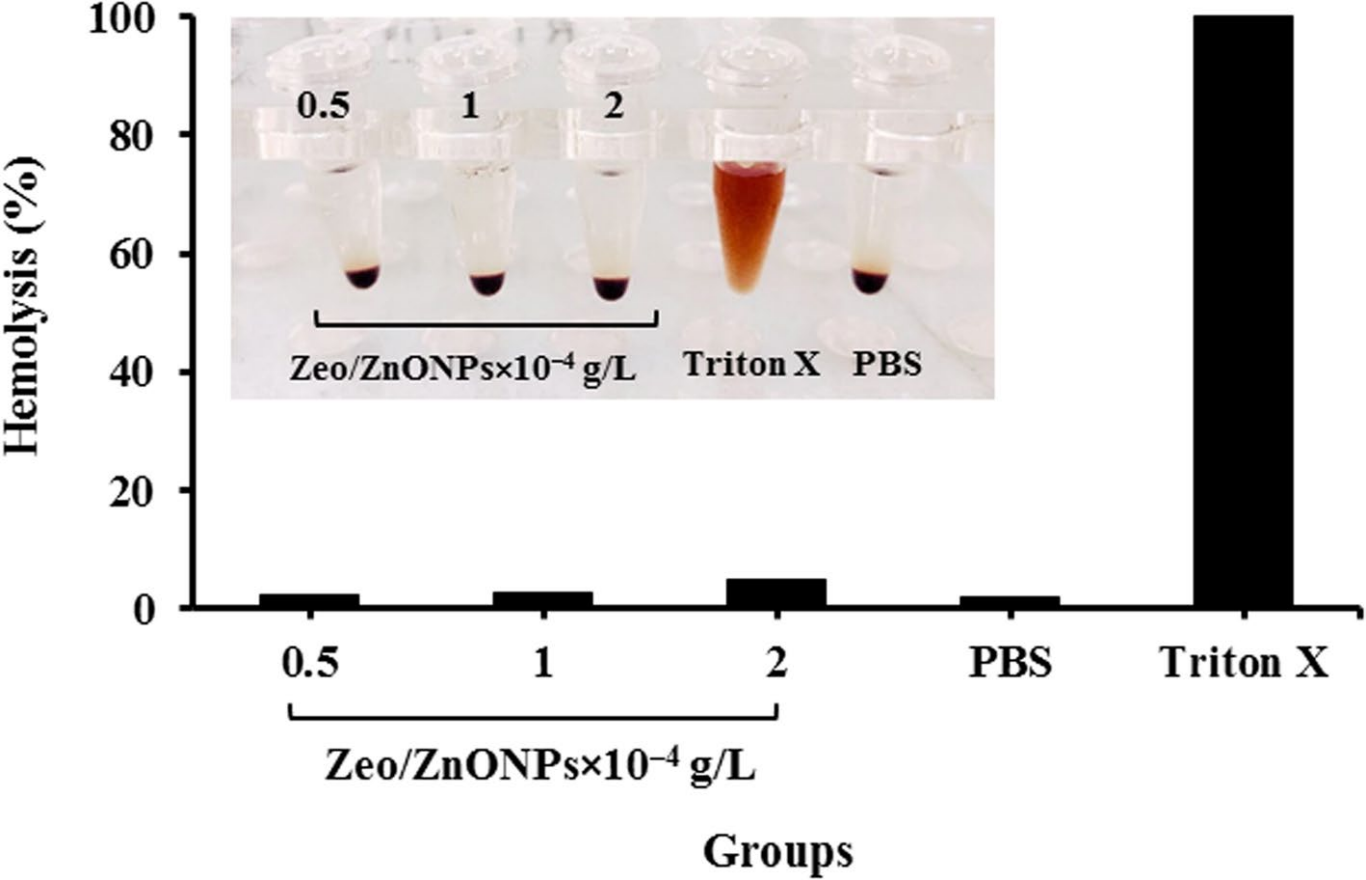
Hemolytic effect of Zeo/ZnONPs

The percentage of hemolytic erythrocytes was assessed to determine whether the synthesized Zeo/ZnONPs destroyed the membrane of RBCs, which can lead to the release of hemoglobin into the plasma by changing membrane integrity and pore formation on RBCs membrane. This evaluation is crucial, as without it the use of Zeo/ZnONPs may result in dangerous pathological conditions. In the current study, the hemolytic activity of Zeo/ZnONPs at the concentrations of 0.5, 1, and 2 × 10^− 4^ g/L exhibited that there was no related destruction of RBCs (Fig. 6), suggesting their great hemocompatibility which was similar to the negative control (PBS).

### MTT and SRB colorimetric assays for cytotoxicity screening

**Fig. 7:**
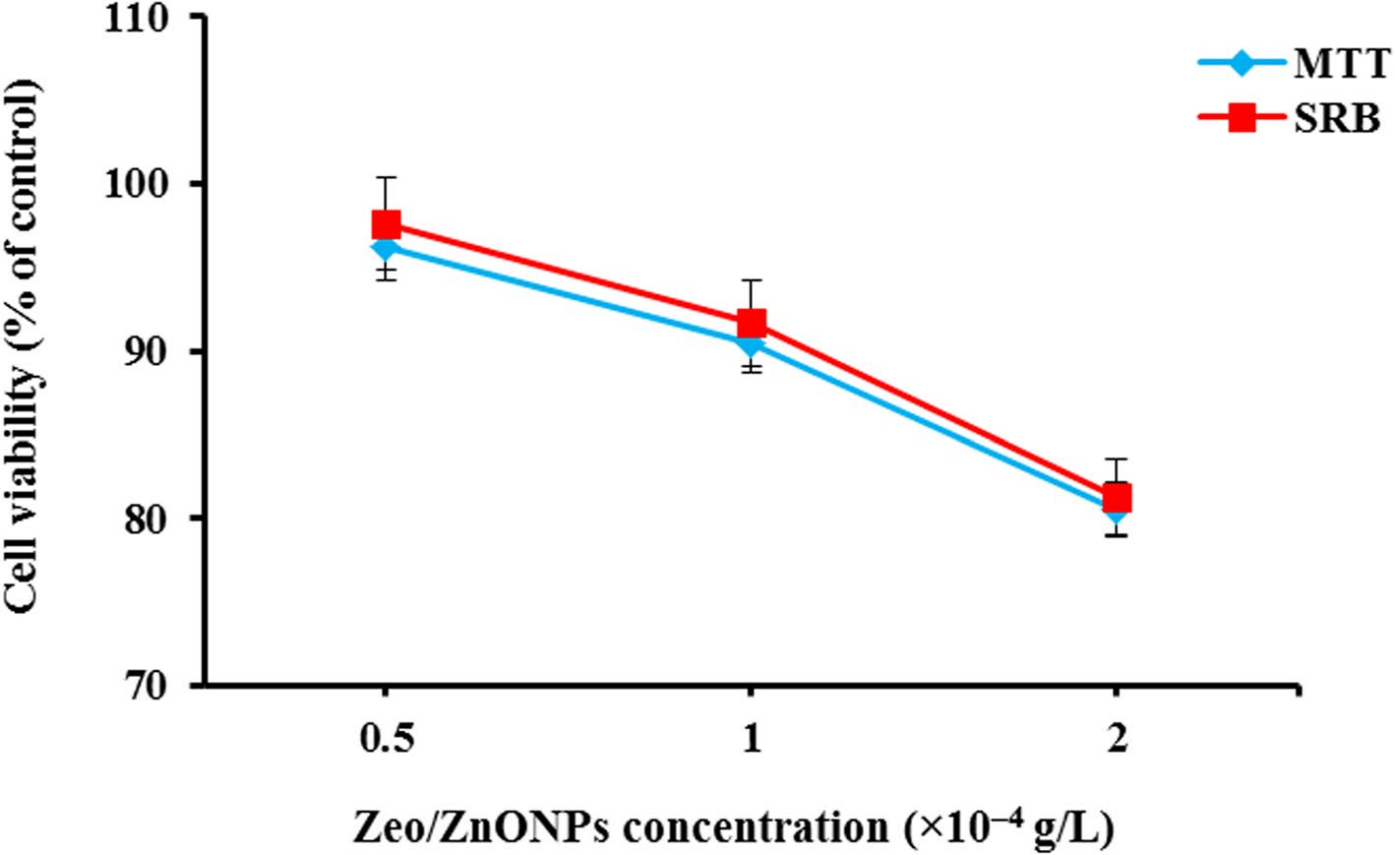
Cytotoxic effects of Zeo/ZnONPs on cell viability of HuGu cells

The cytotoxic effects of Zeo/ZnONPs based on MTT assay on the viability of HuGu cells are presented as percent cell viability in Fig. 7. Treatment with different concentrations of Zeo/ZnONPs did not affect cell viability in HuGu cells, compared with the control group (*P* > 0.05). Also, the results for MTT and SRB showed a similar pattern where there was no considerable decrease in cell viability with increasing Zeo/ZnONPs concentration (P > 0.05). As shown in Fig. 7, the numbers of treated HuGu cells with 2 × 10^− 4^ g/L Zeo/ZnONPs were dropped slightly to 19.4 and 18.7% when compared to the controls in MTT and SRB assays, respectively (*P* > 0.05). Accordingly, the results of the MTT-based cytotoxicity assay were in agreement with the cytotoxic assay based on SRB.

### Intracellular ROS production

**Fig. 8:**
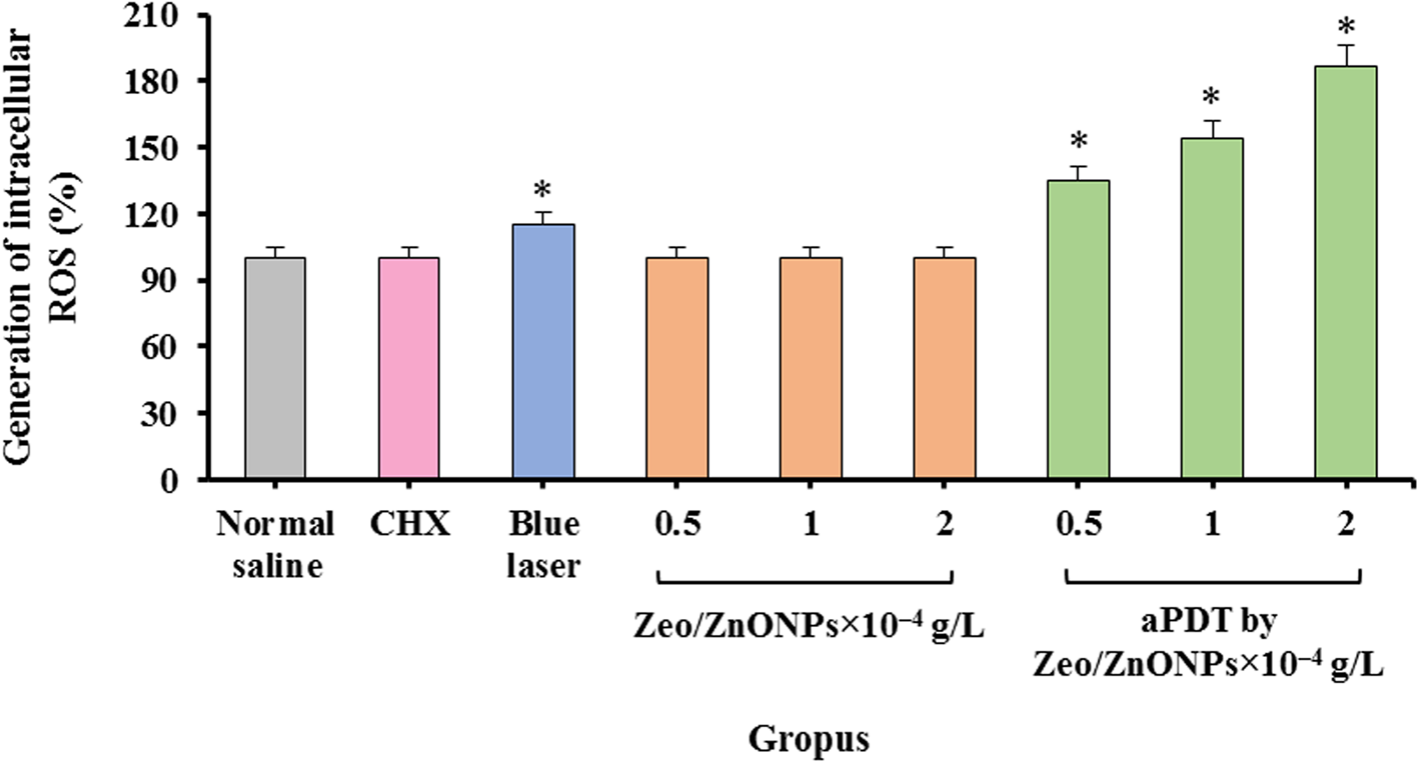
Intracellular ROS production in polymicrobial suspension following different treatments

No significant increase in the DCFH-DA fluorescence was observed in polymicrobial suspension subjected to Zeo/ZnONPs alone and CHX (P > 0.05; Fig. 8). A remarkable increase in the intracellular ROS generation was evident when the polymicrobial suspension was subjected to Zeo/ZnONPs-mediated aPDT, which was dose-dependent (*P* < 0.05). Accordingly, 2 × 10^− 4^ g/L of Zeo/ZnONPs produced 1.87-fold higher ROS in polymicrobial suspension compared to the control group (P < 0.05). Also, the results revealed an increase (1.15-fold) in polymicrobial suspension exposed to blue laser alone.

### Anti-biofilm capability

**Fig. 9:**
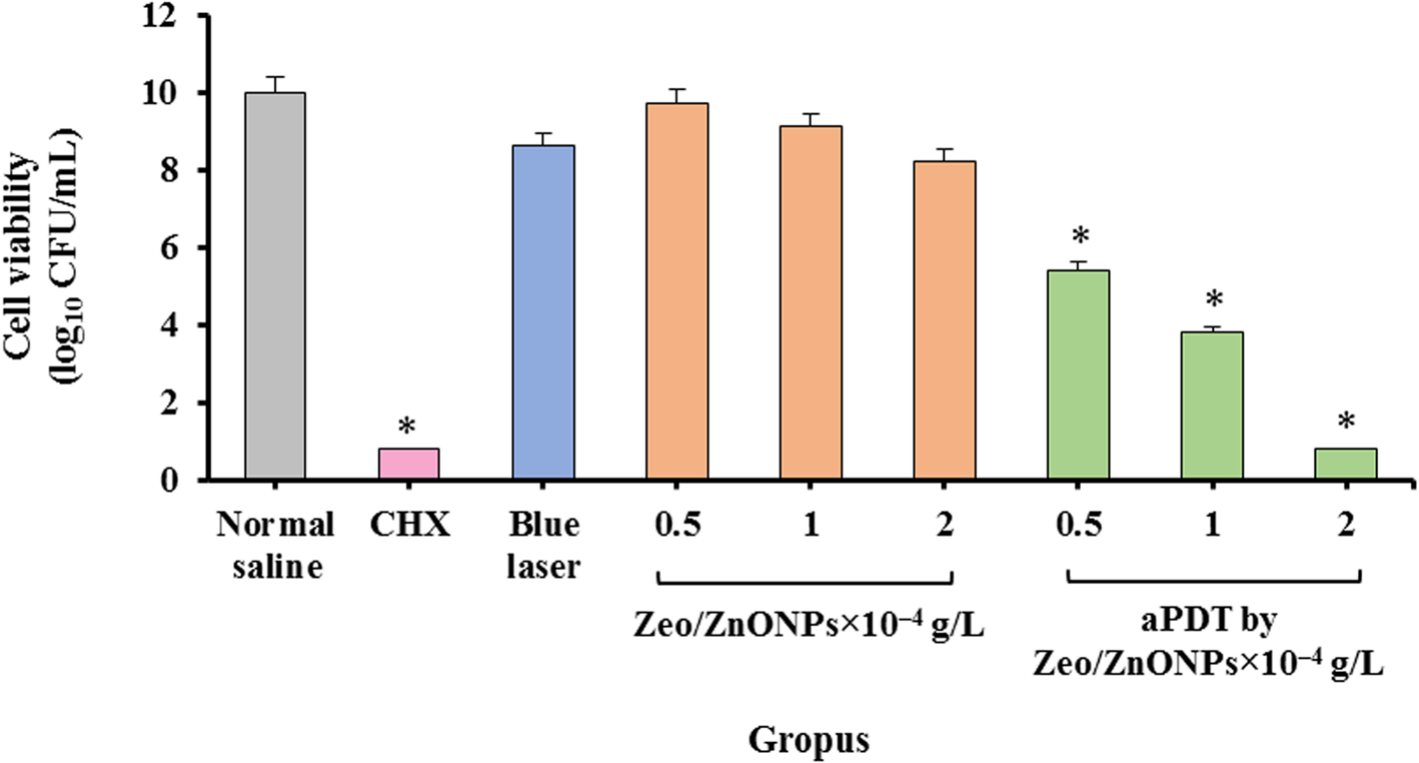
Cell viability of polymicrobial suspension following different treatments

The concentration-dependent anti-biofilm activity was observed in Zeo/ZnONPs (Fig. 9). However, greater anti-biofilm potency was observed by combining Zeo/ZnONPs and blue laser light. When combined, 0.5, 1, and 2 × 10^− 4^ g/L concentrations of Zeo/ZnONPs represent anti-biofilm values of 4.6, 6.2, and 9.2 Log_10_ CFU/mL, respectively. Anti-biofilm activities obtained from both aPDT and 0.2% CHX groups were similar (*P* > 0.05). In contrast, no significant inhibition was achieved from the blue laser alone.

**Fig. 10:**
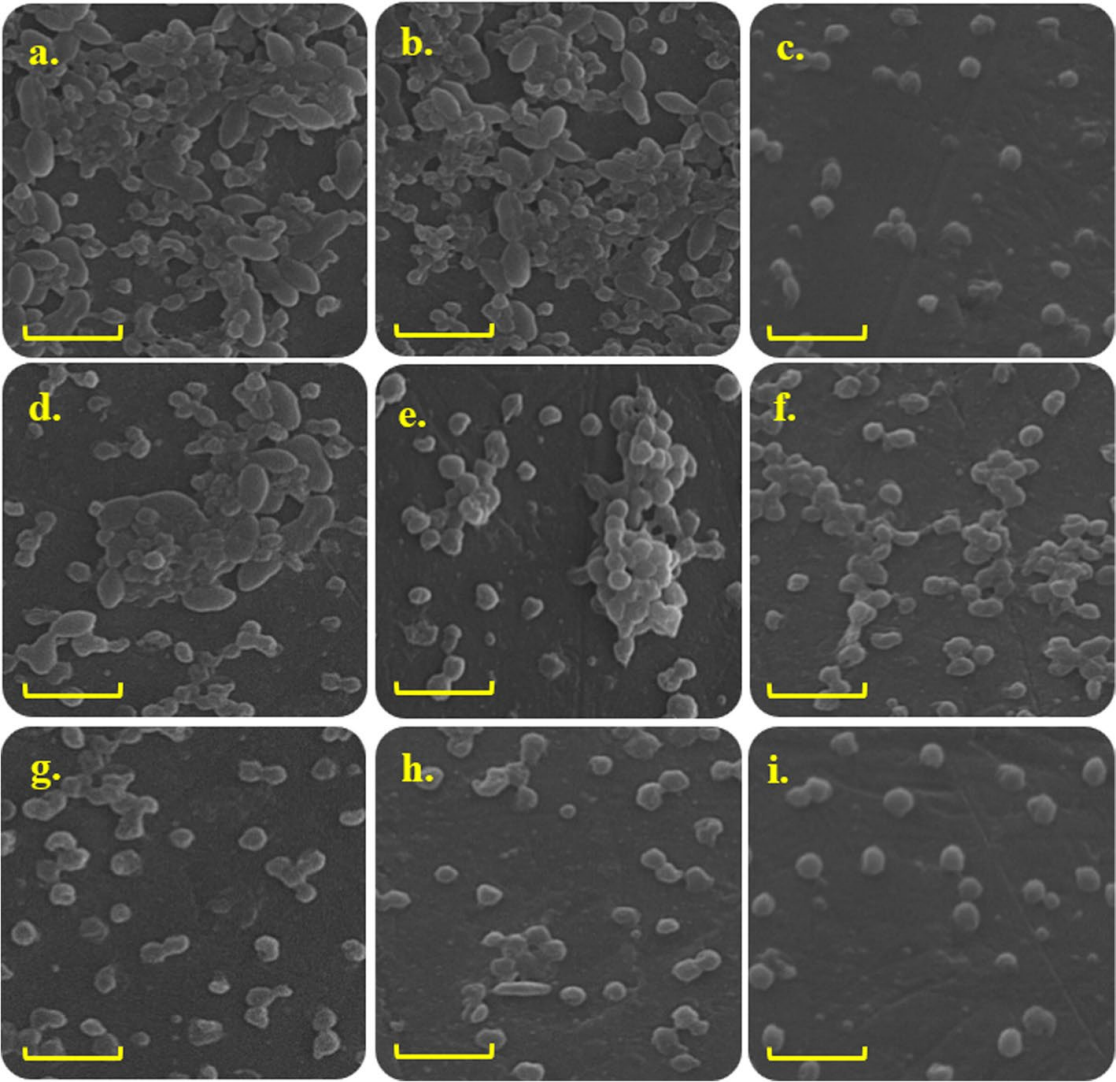
SEM images of polymicrobial biofilms viability following different treatments (Scale bar = 5 μm, Mag = 20.00 KX). a) Negative control: Normal saline, b) Blue laser, c) Positive control:0.2% CHX, d) 0.5 × 10^− 4^ g/L of Zeo/ZnONPs, e) 1 × 10^− 4^ g/L of Zeo/ZnONPs, f) 2 × 10^− 4^ g/L of Zeo/ZnONPs, g) aPDT using 0.5 × 10^− 4^ g/L of Zeo/ZnONPs, h) aPDT using 1 × 10^− 4^ g/L of Zeo/ZnONPs, i) aPDT using 2 × 10^− 4^ g/L of Zeo/ZnONPs

Interestingly, SEM images in Fig. 10 confirm the results obtained from log_10_ CFUs/mL.

### Anti-metabolic activity

**Fig. 11:**
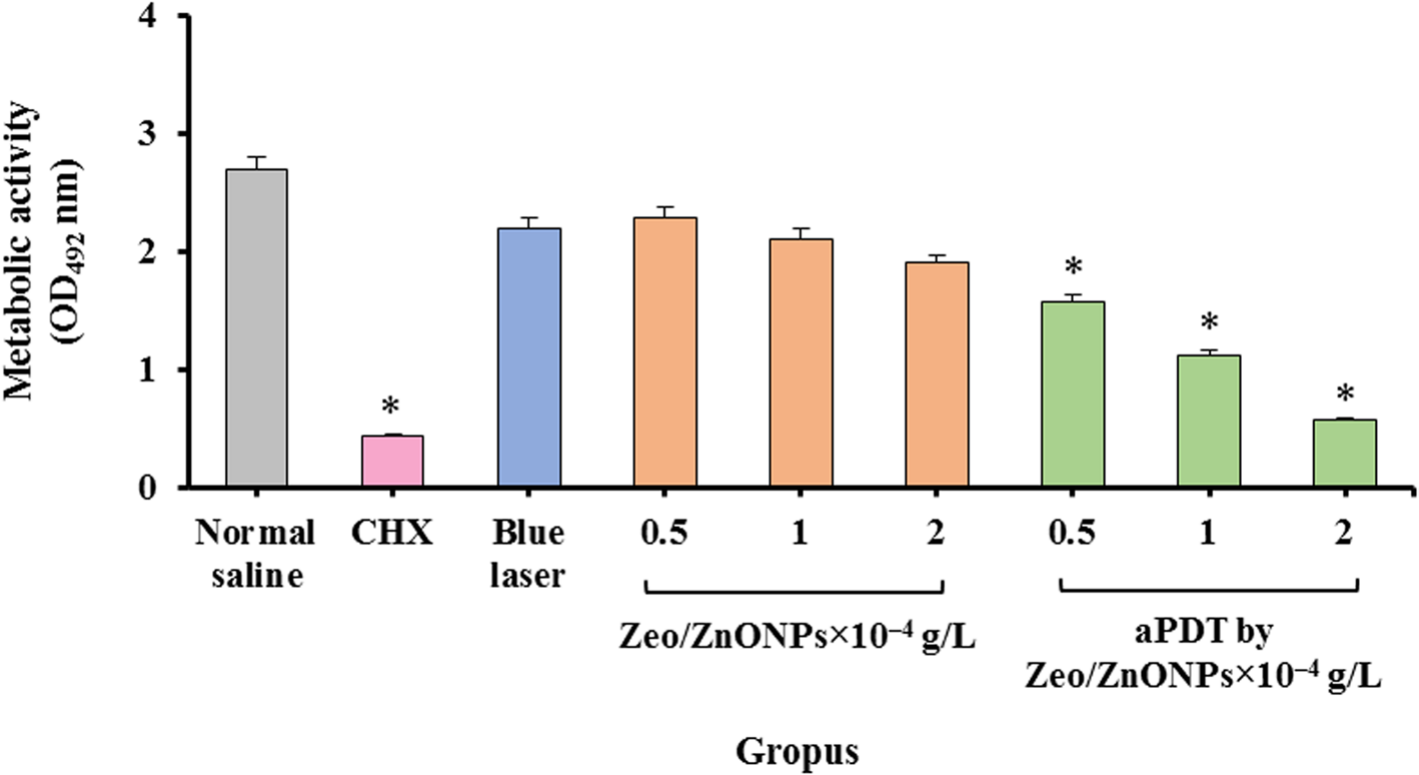
Metabolic activity of polymicrobial suspension following different treatments

The anti-metabolic activity of Zeo/ZnONPs at various concentrations in the combination with blue laser light was evaluated (Fig. 11). According to the XTT colorimetric assay, aPDT using 2 × 10^− 4^ g/L of Zeo/ZnONPs showed significantly greater anti-metabolic activity as compared to the other group with an inhibition percentage of 78.6% (*P* < 0.05). No considerable difference was observed between 2 × 10^− 4^ g/L of Zeo/ZnONPs-medaited aPDT and 0.2% CHX (*P* > 0.05). As shown in Fig. 11, after treatment with 0.5, 1, and 2 × 10^− 4^ g/L of Zeo/ZnONPs, the metabolic activity of polymicrobial biofilms decreased by 15.1, 21.7, and 29.4%, respectively. There was no significant effect of anti-metabolic activity observed of the blue laser light on polymicrobial biofilms (*P* > 0.05).

### Evaluation of enamel remineralization

The results of ESM were shown in Table 1. There was no significant difference between the value of artificial saliva as a control group and that after demineralization (*P* > 0.05). ESM of the NaF varnish group as a treatment control group increased significantly compared with the artificial saliva group (*P* < 0.05). For aPDT group based on 2 × 10^− 4^ g/L of Zeo/ZnONPs, the value of ESM at all time points was consistent with that of NaF varnish group and there was no statistical difference between these two groups (*P* > 0.05). ESM of blue laser and Zeo/ZnONPs groups increased insignificantly comparing with the NaF varnish group (P > 0.05), and there was no statistical difference between those two groups (blue laser and Zeo/ZnONPs groups; P > 0.05).

**Table 1:** Mean of descriptive statistics of enamel microhardness values (EMH) in different conditions.

Treatment	Intact	Demineralized (t_0_)	Demineralized and remineralized	
			t_2_	t_1_
**NaF**	3.78	2.25	3.09	2.57
SD^a^	0.19	0.28	0.17	0.22
Min^b^	3.48	2.02	2.72	2.44
Max^c^	4.15	2.33	3.31	2.76
**0.5 × 10**^**−4**^** g/L of Zeo/ZnONPs**	3.69	2.07	2.18	2.12
SD	0.22	0.19	0.22	0.33
Min	3.31	1.86	2.04	2.01
Max	4.12	2.33	2.51	2.49
**1 × 10**^**−4**^** g/L of Zeo/ZnONPs**	3.64	2.01	2.21	2.09
SD	0.20	0.16	0.31	0.21
Min	3.55	1.84	2.05	1.82
Max	4.01	2.27	2.28	2.16
**2 × 10**^**−4**^** g/L of Zeo/ZnONPs**	3.72	2.18	2.43	2.23
SD	0.18	0.19	0.18	0.25
Min	3.67	2.01	2.25	2.12
Max	3.99	2.64	2.78	2.61
**Blue laser**	3.82	2.28	2.39	2.35
SD	0.19	0.25	0.15	0.24
Min	3.41	2.11	2.23	2.11
Max	3.89	2.40	2.48	2.52
**0.5 × 10**^**−4**^** g/L of Zeo/ZnONPs + Blue laser**	3.73	2.11	2.45	2.22
SD	0.19	0.27	0.22	0.31
Min	3.61	2.01	2.34	2.14
Max	3.86	2.25	2.91	2.64
**1 × 10**^**−4**^** g/L of Zeo/ZnONPs + Blue laser**	3.78	2.25	2.75	2.43
SD	0.26	0.21	0.29	0.27
Min	3.68	2.16	2.27	2.25
Max	4.02	2.47	3.04	2.73
**2 × 10**^**−4**^** g/L of Zeo/ZnONPs + Blue laser**	3.77	2.22	3.42	2.69
SD	0.26	0.27	0.27	0.25
Min	3.31	1.87	2.68	2.33
Max	3.82	2.56	3.58	3.11
**Artificial saliva**	3.88	2.32	2.30	2.36
SD	0.19	0.43	0.21	0.19
Min	3.71	2.02	2.09	2.12
Max	3.93	2.54	2.42	2.54

^a^ Standard deviation

^b^ Minimum

^c^ Maximum

DIAGNOdent Pen results were shown in Table 2. No statistical difference was found among the readings of control, blue laser, and Zeo/ZnONPs groups and with that after demineralization (P > 0.05). Readings of NaF varnish and aPDT groups were at the same level (P > 0.05), which were significantly reduced comparing with the three above-mentioned groups (*P* < 0.05). The reading of aPDT group based on 2 × 10^− 4^ g/L of Zeo/ZnONPs was the lowest, which was statistically different from all of the above-mentioned groups (P < 0.05), except the NaF varnish group.

**Table 2:** Mean of DIAGNOdent Pen readings of the lesions before and after remineralization

Treatment	Intact	Demineralized (t_**0**_)	Demineralized and remineralized	
			t_**1**_	t_**2**_
**NaF**	3.78	9.61	8.37	9.18
SD^a^	0.19	0.71	0.81	0.78
Min^b^	3.48	9.12	8.21	8.91
Max^c^	4.15	9.98	8.83	9.52
**0.5 × 10**^**−4**^ **g/L of Zeo/ZnONPs**	3.69	9.77	9.53	9.67
SD	0.22	0.91	0.61	0.68
Min	3.31	9.57	9.35	8.46
Max	4.12	9.89	9.87	9.81
**1 × 10**^**−4**^ **g/L of Zeo/ZnONPs**	3.64	9.83	9.29	9.64
SD	0.20	0.71	0.92	0.86
Min	3.55	8.33	8.45	9.33
Max	4.01	9.96	9.37	9.84
**2 × 10**^**−4**^ **g/L of Zeo/ZnONPs**	3.72	9.67	8.96	9.41
SD	0.18	0.84	0.61	0.71
Min	3.67	8.73	8.45	8.83
Max	3.99	9.66	9.17	9.60
**Blue laser**	3.82	9.51	8.91	9.26
SD	0.19	0.58	0.82	0.65
Min	3.41	9.45	8.71	9.33
Max	3.89	9.84	9.32	9.78
**0.5 × 10**^**−4**^ **g/L of Zeo/ZnONPs + Blue laser**	3.73	9.69	9.17	9.41
SD	0.19	0.73	0.65	0.79
Min	3.61	9.46	9.01	9.13
Max	3.86	9.88	9.41	9.88
**1 × 10**^**−4**^ **g/L of Zeo/ZnONPs + Blue laser**	3.78	9.60	8.33	8.97
SD	0.26	0.81	0.61	0.76
Min	3.68	8.86	8.04	8.72
Max	4.02	9.79	8.81	9.17
**2 × 10**^**−4**^ **g/L of Zeo/ZnONPs + Blue laser**	3.77	9.58	7.49	7.81
SD	0.26	0.71	0.69	0.81
Min	3.31	9.21	7.25	7.31
Max	3.82	9.87	7.84	8.97
**Artificial saliva**	3.88	9.46	9.48	9.34
SD	0.19	0.72	0.61	0.53
Min	3.71	9.33	9.32	9.55
Max	3.93	9.84	9.91	9.79

^a^Standard deviation

^b^ Minimum

^c^ Maximum

## Discussion

Dental plaque accumulation and inadequate personal oral hygiene are the severe challenges for maintaining oral health in orthodontic patients [26]. Reduction of bacterial colonization and removal of dental biofilms as one of the most important etiologic parameters for oral pathological conditions are performed by various techniques, but with limited success [27, 28].

Recently, aPDT has been introduced as an alternative therapy for preventing and treating dental caries via control the formation of the microbial biofilm and controlling the incidence of microbial pathogens without the development of resistance [29–31]. Several variables such as the type and concentration of photosensitizers, light sources, irradiation parameters, and physiologic condition of the microorganisms studied must be considered for the effectiveness of aPDT [32].

Many in vitro and in vivo studies present high efficiency of aPDT against microorganisms involved in dental caries [13, 33–37]. The results of Alqerban [38] study showed that not only aPDT with Riboflavin and Rose Bengal can be used for bonding orthodontic brackets to the tooth surface but also revealed the substantial antibacterial properties against *S. mutans*. Hugo Panhóca et al. [39] reported aPDT with Curcumin reduces the number of living cells of *S. mutans* in a biofilm model created on the surface of metallic orthodontic accessories.

The results of previous studies showed that ZnONPs are the most promising next-generation photosensitizer for PDT due to their specific phototoxic effect on tumor areas [40–43]. According to Pourhajibgher et al. photo-activated Curcumin doped ZnONPs can use as an orthodontic adhesive additive to control the cariogenic multispecies biofilm, and also reduce their metabolic activity [7].

The biocidal cations of ZnONPs have been hosted on the surfaces and in the cavities of Zeo via ion exchange and can become an efficient nano-photosensitizer in aPDT process. On the other hand, Zeo as stable carriers with high chemical inertness and null toxicity could be increasing the efficacy of photosensitizer via protecting it from oxidation, increasing the permeability of the photosensitizer through the cell membrane, increasing the concentration of photosensitizer molecules in the target cells, and increasing the lifetime of photosensitizer release through a sustained process [44–47].

The possible risks of nano-photosensitizer to human health have raised concerns. These concerns underline the need and importance of assessing their cytotoxicity. In a work reported by Wang et al. [48] the cytotoxic effects of ZnONPs at concentrations of 10, 15, 30, and 100 μg/mL was investigated on different cell types such as human keratinocyte cells (HaCaT), human gingival fibroblast (HGF-1), and human gingival squamous carcinoma cell line (Ca9–22). Their results showed that ZnONPs were less toxic to normal HaCaT and HGF-1 cells, but showed severe toxicity to Ca9–22 cells at concentrations more than 30 μg/mL. Seker et al. [49] and Vergara-Llanos et al. [50] reported ZnONPs exhibited cytotoxic effect at doses of 50–100 μg/mL. Since there has been no report so far of using SRB dye in testing cytotoxicity of photosensitizers, and also considering advantages of the SRB assay such as safety and high stability of staining, in this study, the cell-killing effect of Zeo/ZnONPs as the model photosensitizer at the different concentrations was tested on HuGu cells. Zeo/ZnONPs at 0.5 × 10^− 4^ g/L concentration had very low cytotoxicity; 3.8 and 2.4% of HuGu cells are viable following MTT- and SRB-based cytotoxicity assay, respectively. In recent years, the MTT assay has been the most widely used; however, it still has a few disadvantages. For example, the amount of MTT is not linear with cell concentration at high cell number, cell lines differ in their activity to reduce the MTT dye and the assay has large inter-assay and intra-assay variations under several conditions. Some MTT reagents also are suspected of causing genetic defects. Although there has been no report concerning physical hazards in using MTT reagents, the handling storage, and disposal of MTT reagents pose safety and environmental concerns. Thus, MTT waste must be appropriately eliminated after testing. Recently, the SRB protein staining was described as an alternative approach. The SRB assay appeared to be more sensitive than the MTT assay, with higher reproducibility and better linearity with cell concentration. In contrast to the MTT assay, SRB staining is stable and plates can be stored up to several months. SRB assay has the advantage of avoidance of handling bio-hazard compounds [51]. According to the excellent agreement was noted for the evaluation of Zeo/ZnONPs cytotoxicity and also regarding advantages of the SRB assay, this method can be used for cytotoxic screening of Zeo/ZnONPs.

Also, after a 24 h incubation period, 19.4 and 18.7% of HuGu cells were viable at the highest concentration of Zeo/ZnONPs (2 × 10^− 4^ g/L) following MTT and SRB assay, respectively. As the results displayed the cytotoxicity of Zeo/ZnONPs was dependent on concentration. In this study, the hemolytic effect of Zeo/ZnONPs on erythrocytes was assessed and its biocompatibility at the different concentrations was revealed. Babu et al. [52] explored the hemolytic effect of ZnONPs was increased with increasing concentration from 25 to 800 μg/mL in a time-dependent manner. Besides, the data from Ulyanova et al. [53] showed that with a decrease in the concentration of nano-zeo to 1 mg/mL, there is a tendency for the hemolytic activity of the samples to decrease. Therefore, the results obtained from this study are consistent with the results of previous studies.

There are no studies in the literature on using Zeo/ZnONPs- mediated aPDT for the inhibition of polymicrobial biofilms growth in patients undergoing orthodontic treatment. Herein, a significant decrease on the viability of microorganisms was observed when biofilms were exposed to aPDT. The present study showed an interesting result with 2 × 10^− 4^ g/L of Zeo/ZnONPs- mediated aPDT, degradation 92 and 78.6% of biofilms, and metabolic activity of polymicrobial biofilms, respectively. In addition, no remarkable difference in the number of log_10_ CFU/mL was observed between Zeo/ZnONPs- mediated aPDT (2 × 10^− 4^ g/L) and CHX.

Following light irradiation, the photosensitizer in its ground singlet state excited and reacts with oxygen to produce ROS. These ROS can be classified into those produced by Type I photochemical mechanism: free radicals such as superoxide radical anion (O2^•−^) and hydroxyl radicals (HO^•^) and those produced by Type II photochemical mechanism: singlet oxygen (^1^O_2_). They all have damage effects on biomolecules and kill the target cells, resulting in membrane destructive process, metabolic hydroxylation, oxidative DNA damage, carcinogenesis, etc. [54]. As Zhang et al. [55] reported, the production of ROS is dependent on photosensitizer concentration, as well as, irradiation dose of the light source. In this ex vivo study, the results demonstrate that 2 × 10^− 4^ g/L Zeo/ZnONPs can generate ROS within polymicrobial biofilms after blue laser irradiation about 1.87-fold.

To the best of our knowledge, this is the first time to evaluate the remineralization potential of enamel carious lesions. Enamel samples treated with Zeo/ZnONPs-based aPDT exhibited enhanced surface microhardness when compared with samples treated with artificial saliva alone. In this study, like several previous studies, the NaF varnish was effective for enamel remineralization [56–58]. However, aPDT using 2 × 10^− 4^ g/L Zeo/ZnONPs and the NaF varnish groups were not statistically different (*P* > 0.05).

## Conclusion

In summary, the results of this study have proved that Zeo/ZnONPs-based aPDT has emerged a promising potential as an adjuvant therapeutic approach in the fields of anti-biofilms and anti-caries, which are involved with their potent ability to produce ROS. On the other hand, the current results suggest that aPDT using Zeo/ZnONPs could have the greatest remineralization efficacy to improve the microhardness of the enamel surface after microbial demineralization.

## Materials and methods

### Statement

In the current study, all methods and experiments were carried out in accordance with relevant guidelines and regulations.

### Synthesis of Zeo\ZnONPs as the photosensitizer

Zeo\ZnONPs was prepared as previously described by Alswata et al. [59] with a slight modification. Briefly, 5 g of Zeo powder (Sigma-Aldrich, United Kingdom) was dissolved into 100 mL deionized water to form a solution. 3 g of Zn (NO_3_)_2_·4H_2_O (98.5%; obtained from Merck, Germany) was then added to the suspension. The mixture was stirred under reflux reaction at 80 °C for 3 h. 2 M of sodium hydroxide (NaOH) of 99% (Merck, Germany) was dropped into the solution for precipitation of ZnO onto the Zeo. The suspension was re-stirred until pH 11 was adjusted and the color changed to black. After that, the black product was filtered, washed three times with deionized water, and dried at 80 °C for 12 h. After drying, the final product was obtained after annealing at 450 °C for 2 h.

### Characterization of Zeo/ZnONPs

#### Field emission scanning electron microscopy (FESEM)

The surface morphology of Zeo/ZnONPs was studied by FESEM (ZEISS, German) under the voltage of 15 kV.

#### Transmission electron microscope (TEM)

The transmission electron microscope (TEM; Zeiss EM10C) with an accelerating voltage of 80 Kv was used to assess the particle size and size distribution of Zeo\ZnONPs.

#### Dynamic light scattering (DLS) and zeta potential analysis

The size distribution profiles of nanometer-sized particles in suspension and Zeta potential measurements were carried out using a MALVERN Zetasizer Ver. 6.01 (Malvern Instruments, UK) at approximately 25 °C.

#### Absorption spectrum

The absorption spectrum of synthesized Zeo/ZnONPs was carried out by UV-visible spectrophotometer, scanning the absorbance spectra in the range of 350–500 nm wavelength.

#### Mapping materials

Energy dispersive spectroscopy (EDS) mapping was employed to confirm the presence of chemical elements in the structure of Zeo/ZnONPs.

### Hemolytic activity of Zeo/ZnONPs

The hemolysis experiment was performed to test the biocompatibility of the synthesized NET according to Pourhajibagher et al. study [60]. In summary, after the collection and addition of sodium citrate to 2 mL of fresh human blood samples obtained from returned unused blood bag in blood bank (Iranian Blood Transfusion Organization), they were washed and centrifuged at 1000 rpm for 10 min to get the red blood cells (RBCs) as a pellet. The pellet was re-suspended with phosphate-buffered saline (PBS) and treated with an equal volume of synthesized Zeo/ZnONPs at different concentrations (0.5, 1, and 2 × 10^− 4^ g/L). 2% Triton X and PBS buffer were used as the positive and negative controls, respectively. Following incubation of the samples at 25 °C for 30 min, RBCs were collected as a pellet by centrifugation at 2000 rpm for 3 min. The supernatant was utilized to evaluate the absorbance at 540 nm using a microplate reader. The percentage of hemolysis was then calculated using the following equation:

$$\mathrm{Hemolysis}\ \left(\%\right)=\frac{\mathrm{OD}\ \mathrm{test}\ \mathrm{sample}-\mathrm{OD}\ \mathrm{PBS}\ }{\mathrm{OD}\ \mathrm{Triton}\ \mathrm{X}-\mathrm{OD}\ \mathrm{PBS}}\times 100$$

### Cytotoxicity evaluation of Zeo/ZnONPs

#### Cell culture procedure

Human gingival fibroblast cells (HuGu; IBRC C10459) obtained from Iranian Biological Resource Center, Tehran, Iran were seeded in a 96-well plate at a plating density of 4 × 10^4^ cells/well. The cells were maintained in 100 μL DMEM containing 10% fetal bovine serum (FBS) solution and antibiotics (200 μL/mL penicillin G, 200 μg/mL streptomycin, and 2 μg/mL fungizone) at 37 °C, 5% CO_2_ 95% air, and 100% relative humidity. After 24 h, Zeo\ZnONPs at the concentrations of 0.5, 1, and 2 × 10^− 4^ g/L were added into the wells and the well-plate was incubated for 24 h at 37 °C, 5% CO_2_, and 95% air with 100% relative humidity.

#### Tetrazolium (MTT)-based cytotoxicity assay

The cell viability was evaluated using a modified 3-(4, 5-dimethylthiazol-2-yl)-2, 5-diphenyl tetrazolium (MTT) assay. The solutions were removed from each well and 50 μL of MTT reagent (5 mg/mL) was added and the well-plate was incubated for 4 h at 37 °C in the CO_2_ incubator. The MTT solution was then discarded, 100 μL of dimethyl sulfoxide (DMSO) was added to dissolved the formed purple crystal formazan, and the absorbance was measured at a wavelength of 570 nm using a microplate reader. DMEM prepared without the addition of samples were used as the control. Percentage cell viability was determined using the following equation:

$$\mathrm{Cell}\ \mathrm{viabiliy}\ \left(\%\right)=\frac{\mathrm{Absorbance}\ \left(\mathrm{sample}\right)\ }{\mathrm{Absorbance}\ \left(\mathrm{Control}\right)}\times 100$$

#### Sulforhodamine B (SRB)-based cytotoxicity assay

The SRB assay was used to investigate cytotoxicity in cells based on the measurement of cellular protein content [60]. After 24 h incubation, HuGu cells were fixed with 10% (wt/vol) trichloroacetic acid for 30 min. The cells were then washed repeatedly with 1% (vol/vol) acetic acid to remove the excess dye. 50 μL of SRB solution (0.04% [wt/vol]; Merck, Germany) was added to each well and the well-plate was incubated at room temperature. After 1 h, the wells were washed four times with 1% (vol/vol) acetic acid to remove unbound dye. Finally, 200 μL of 10 mM Tris base solution (pH 10.5) was used to dissolve the protein-bound dye and OD was determined at a wavelength of 510 nm using a microplate reader. Percentage cell viability was determined similar to the MTT assay.

### Microorganisms and growth conditions

Standard strains of *Streptococcus mutans* ATCC 35668, *Lactobacillus acidophilus* ATCC 314, and *Candida albicans* ATCC 10231 obtained from Iranian Biological Resource Center, Tehran, Iran were cultured in Brain heart infusion (BHI) broth (Merck, Germany) and incubated at 37 °C in a shaker incubator at 150 rpm to achieve an optical density (OD) at a wavelength of 600 nm between 0.08–0.13, in the same range as equivalent to that of the 0.5 McFarland standard.

### Light source

A blue laser (Laser Diode Stabilizer LDS201, Asha beam profile, Iran) in a continuous beam was used as a light source with an output intensity of 150 mW/cm^2^, 4.2 V, and 0.34 A at the wavelength of 405 ± 10 nm. A photodiode power meter (PMB- 104 power meter, Asha beam profile, Iran) was utilized to measure the output power at the optic tip. ​All the steps from the operatory protocol were set according to the manufacturer’s recommendations.

### Enamel slab preparation

Human premolars without visible cracks, enamel irregularities, and WSLs in the buccal and lingual enamel surfaces which extracted for orthodontic purpose were selected. All teeth experiments have been approved by the Ethics Committee of Tehran University of Medical Sciences (Application No. IR.TUMS.MEDICINE.REC.1400.52106), and the need for informed consent was waived by the ethical committee. All the teeth were disinfected and stored in thymol solution (0.1%) at 4 °C before use.

Enamel slabs (approximately 3 × 3 × 1 mm) were prepared from mid-labial tooth parts by excision using a water-cooled carborundum disc. For removal of about a 100 μm depth of enamel, the flat surface of slabs was then polished with diamond paste (water-based 0.25-μm diamond particles). Finally, the prepared enamel slabs were ultrasonically cleaned for 15 min.

### Specimen preparation for microbial experiments

**Fig. 12:**
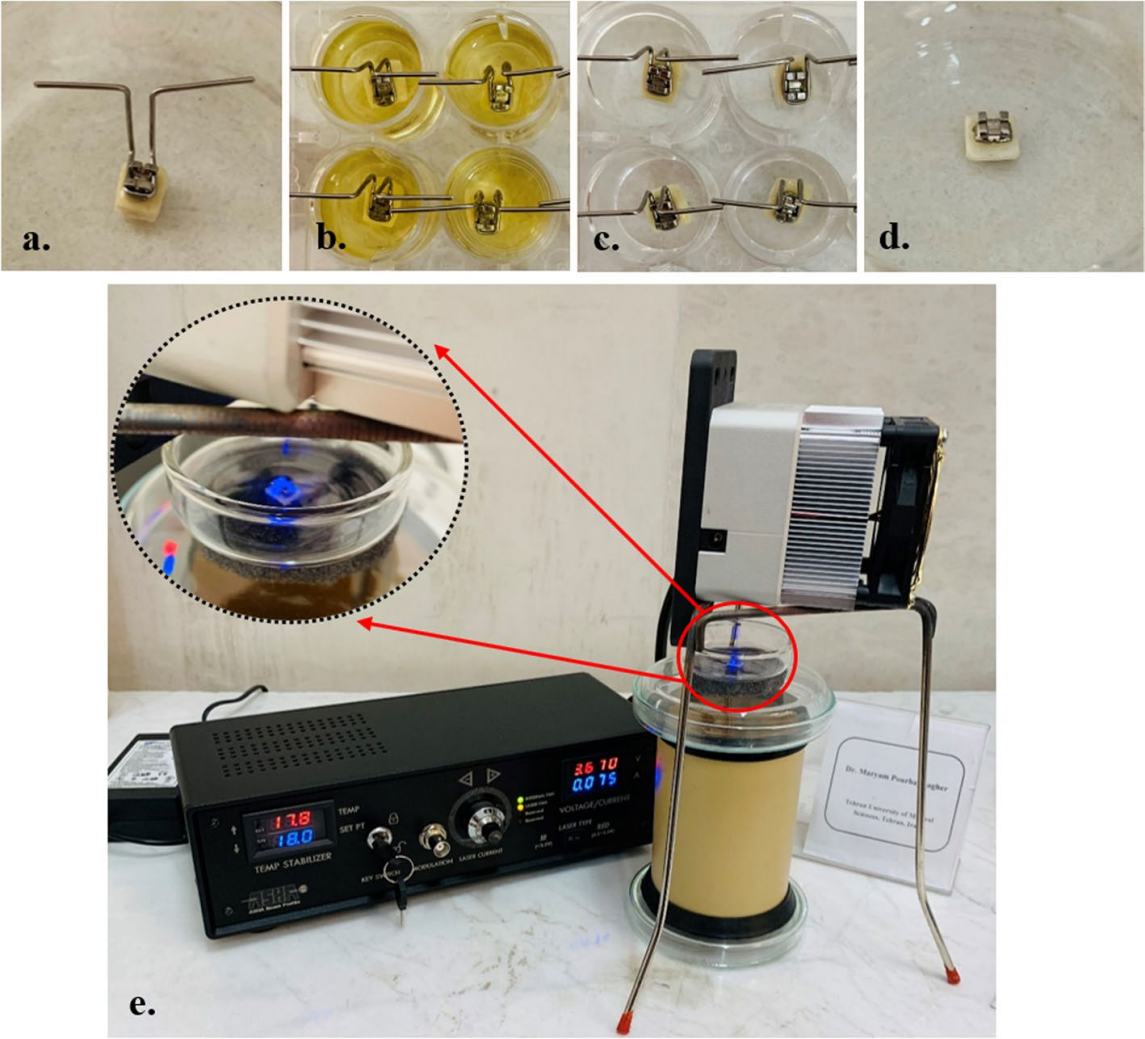
Treatment process; **a** Fabrication of enamel slab bonded bracket attached to the stainless-steel orthodontic wires, **b** Immersion of the enamel slab bonded bracket in wells of the microplate containing growth medium to formation of microbial biofilms on enamel slab bonded bracket, **c** Rinsing of the enamel slab bonded bracket with sterile saline to eliminate the planktonic and weak attached microorganisms, **d** Treatment of enamel slab bonded bracket with different concentrations of Zeo/ZnONPs, and **e** Irradiation of treated enamel slab bonded bracket with a blue laser light

Prepared enamel slabs were etched with the use of 37% phosphoric acid gel for 30 s, rinsed, and dried for 10 s. A thin layer of a liquid resin primer (Transbond XT primer, 3 M Unitek, Orthodontic Products, Monrovia, USA) was brushed on the etched enamel surface and light-cured with the visible light-curing for 20 s. The composite resin was placed onto the titanium orthodontic bracket (Parmis Teb, Isfahan, Iran) and the bracket was bonded on the enamel surface with a consonant force and light-cured for 30 s. The brackets were bonded on the metallic supports made with stainless steel orthodontic wire to suspended the samples for more accumulation of microbial biofilms (Fig. 12 a-c). Before the experiments, enamel slabs with bonded brackets and the metallic supports were sterilized using 4.08 kGy of gamma radiation and stored in a humid atmosphere at 4 °C.

### Formation of polymicrobial biofilms on the orthodontic brackets

The orthodontic brackets were placed in the 24-well plates (Ningbo Fuchun, China) with 1.5 mL BHI broth supplemented with 5% sucrose, and inoculated with 100 μL of each bacterial suspension at the concentration of 0.5 McFarland standard. The orthodontic brackets were incubated at 37 °C for 72 h under capnophilic conditions (5% CO_2_) for the formation of polymicrobial biofilms [61]. After this time, the brackets were rinsed with PBS (pH 7.4) for removing non-adhered microorganisms. Eventually, the stainless-steel orthodontic wires were detached (Fig. 12 d) and the preformed polymicrobial biofilms on the orthodontic bracket were treated according to the study design (Fig. 12 e).

### Experimental design

The orthodontic brackets were randomly divided into the experimental groups with 10 samples in each group by Random Allocation Software based on the selected type of blocking as follow:

A. Zeo/ZnONPs.
B. Blue laser.
C. aPDT.
D. Positive control: chlorhexidine (CHX).
E. Negative control: Normal saline.

### Treatment procedure

- **Group A:** 100 μL of Zeo/ZnONPs at the concentrations of 0.5, 1, and 2 × 10^− 4^ g/L was added separately to the orthodontic brackets containing polymicrobial biofilms and the samples were incubated in the dark at room temperature for 5 min.
- **Group B:** Polymicrobial biofilms on the orthodontic brackets were exposed with blue laser irradiation at the wavelength of 405 ± 10 nm for 1 min and output intensity of 150 mW/cm^2^. The optical fiber reached up to 2 cm shorter than working length.
- **Group C:** Polymicrobial biofilms on the orthodontic brackets were treated by Zeo/ZnONPs similar to group A and the samples were then exposed with a blue laser similar to group B.
- **Group D:** 100 μL of 0.2% CHX was added to the orthodontic brackets and the samples were incubated at room temperature for 5 min.
- **Group E:** 100 μL of normal saline was added to the orthodontic brackets and the samples were incubated at room temperature for 5 min.

### Detection of intracellular ROS production

The production of ROS by microorganisms involved in polymicrobial biofilms after treatment with different groups was evaluated using 2′-7′-dichlorodihydrofluorescein diacetate (DCFH-DA) (Sigma-Aldrich, United Kingdom) as described by Pourhajibagher et al. [62]. In summary, 100 μL of 5 μM DCFH-DA was added to the mixture (100 μL) of the microorganisms involved in polymicrobial biofilms and shaken at 37 °C for 10 min. The microbial cells were then centrifuged at 5000 rpm for 15 min and the pellets were rinsed with PBS. The cleaned microbial cells were treated based on the experimental design section and the fluorescence emission of DCFH-DA was measured at 535 nm using a microplate reader with an excitation wavelength of 488 nm.

### Evaluation of anti-biofilm potency

After treatment of polymicrobial biofilms on the orthodontic brackets according to the experimental design described, each bracket was transferred to a sterile 5 mL eppendorf containing 2 mL of PBS and was sonicated at a frequency of 20 kHz and output power of 5 W for 20 s to remove the remaining polymicrobial biofilms from the surface of the orthodontic bracket. Then, the serial dilutions were prepared, and 10 μL of each dilution was cultured onto BHI agar plates. The plates were incubated at 37 °C for 24 h under capnophilic conditions and log_10_ CFUs/mL was counted based on the previous study [63]. As well as, the anti-biofilm activities of study groups were assessed by SEM.

### Evaluation of anti-metabolic activity

As Coraça-Hubér et al. [64] study, the metabolic activity was assessed using the XTT (2,3-bis [2-methyloxy-4-nitro-5-sulfophenyl]-2H-tetrazolium-5-carboxanilide) reduction assay (Roche Applied Science, Indianapolis, IN, US). Following sonication (a frequency of 20 kHz and output power of 5 W for 20 s) of treated orthodontic brackets based on the experimental design section, the microbial suspensions were centrifuged at 2000 rpm for 10 min. The supernatants were removed and microbial cell sediments were dissolved in 150 μL of XTT-menadione-PBS solution in 96-well plates and incubated at 37 °C for 3 h. 100 μL of the solution was then transferred to a new 96-well plate and the OD was measured at 492 nm using a microplate reader.

### Polymicrobial biofilm induced enamel demineralization/ treatment induced remineralization assays

Prepared enamel slabs with a microhardness ranging from 2.52 to 3.09 gigapascals (GPa) were used for multi-species biofilm induced enamel demineralization and treatments induced remineralization assays. For these purposes, enamel slabs were separately transferred aseptically into a sterile 24-well tissue culture plate containing 500 μL of sterile artificial saliva and incubated 1 h at room temperature (22 ± 2 °C). After that, the saliva was discarded and 1.5 mL of BHI medium containing 1% sucrose as the growth medium were added. Plates were incubated in 5% CO_2_, for up to 10 days at 37 °C. The growth medium was replaced every 48 h. At the end of the experimental periods, enamel slabs were washed for 10 s in sterile PBS and transferred to microtubes for enamel demineralization using DIAGNOdent Pen reading and microhardness assays. In this study accepted range of microhardness for enamel demineralization was defined as the previous study (2–3 GPa) [55–57], the demineralized samples would be sorted into distill water for enamel remineralization assays using DIAGNOdent Pen reading and microhardness assays.

### Evaluation of the treatment effects on enamel remineralization

Enamel demineralized samples distributed randomly into 7 groups (*n* = 10) as follows:

A. Remineralization effect of Zeo/ZnONPs.
B. Remineralization effect of blue laser.
C. Remineralization effect of aPDT at three concentrations of Zeo/ZnONPs (0.5, 1, and 2 × 10^− 4^ g/L).
D. Remineralization effect of sodium fluoride (NaF) varnish as treatment-control group.
E. Remineralization effect of artificial saliva as the negative control group.

The treatment duration times were 1 month (t_1_) and 3 months (t_2_). Enamel remineralization was evaluated based on the following assays:

#### Diagnodent pen reading

The surface change presented on each experimental enamel slabs after treatment was evaluated at baseline (t_0_) and at the end of treatment duration times (t_1_ and t_2_) using Type B probe of DIAGNOdent Pen 2190 (Kavo, Biberach, Germany) as recommended by the manufacturer guideline. In this assay, NaF varnish and artificial saliva also stood for the treatment-control group and the negative control group, respectively. The experiment was performed in triplicate and the mean value was calculated.

#### Surface microhardness measurement

After reading the treated enamel slabs using DIAGNOdent Pen, the slabs were used to measurement of surface microhardness. Enamel surface microhardness (ESM) was assessed using a digital hardness testing machine (FM-700, Future Tech, Tokyo, Japan) as described previously [65]. The mean ESM measurement value of three indentations at intervals of 0.1 mm which conducted on the surface of each enamel slabs after treatment using a square-based diamond pyramid Vickers’s indenter (at a load of 50 g for 15 s) was calculated at baseline (t_0_) and the end of treatment duration time.

### Data analysis

All assays were done in triplicate and the data were represented as mean values with standard deviation (± SD). The results were statistically evaluated by one-way analysis of variance (ANOVA) and Tukey test. A value of *P* < 0.05 was considered statistically significant.

## Data Availability

All data of this manuscript are included in the manuscript. All figures are original images and have been used for the first time in this study Any additional information required will be provided by communicating with the corresponding author via the official mail: abahador@sina.tums.ac.ir.

## Abbreviations

aPDT: Antimicrobial photodynamic therapy
BHI: Brain heart infusion
CHX: Chlorhexidine
NaF: Sodium fluoride
ROS: reactive oxygen species
Zeos: Zeolites
Zeo/ZnONPs: Zeolite-zinc oxide nanoparticles
ZnONPs: Zinc oxide nanoparticles
WSLs: White spot lesions
